# Supplementary material for: Tight-binding approach to penta-graphene
Source: Sci Rep. 2016 Mar 4;6:22672. doi: 10.1038/srep22672 (PMC4778137; doi:10.1038/srep22672)
Supplement: Supplementary Information [file srep22672-s1.pdf]

Supplementary Information to ”Tight-binding approach to penta-graphene”

T. Stauber[1,*], J. I. Beltrán[1,2,3], and J. Schliemann[4]

[1]Instituto de Ciencia de Materiales de Madrid, CSIC, 28049 Madrid, Spain
[2]GFMC and Instituto Pluridisciplinar, Departamento de Física Aplicada III, Universidad Complutense de Madrid, 28040 Madrid, Spain
[3]IMDEA Materials Institute, C/Eric Kandel 2, 28906 Getafe, Madrid, Spain
[4]Institute for Theoretical Physics, University of Regensburg, D-93040 Regensburg, Germany
[*]tobias.stauber@csic.es

ABSTRACT

In this Supplementary Information, we discuss correlation effects on the band structure when sp^3-hybridized carbon atoms are included, i.e., the onsite Hubbard interaction and assisted hopping terms. We also calculate the optical conductivity using first principles.

Effective Hamiltonian including correlation effects

In order to adequately describe the conduction band of penta-graphene, the C1-atoms as well as correlation effects need to be included. The minimal model consists of one (effective) p_z-orbital for each carbon atom. Additionally, a Hubbard interaction at the C2-atoms as well as an assisted hopping term is included where the hopping amplitude depends on the occupation number of the C2-atoms.[1,2] On the other hand, we neglect the onsite interaction at the C1-atoms because the effective p_z-orbital is spread out due to the sp^3-hypridization. The effective model thus reads

$$\mathscr{H} = \mathscr{H}_0 + \mathscr{H}_C + \mathscr{H}_H + \mathscr{H}_a \tag{1}$$

where we have defined the bare hopping Hamiltonian

$$\begin{aligned}\mathscr{H}_0 = &-t_0 \sum_{m,n;\sigma} \left(a^\dagger_{m,n;\sigma} b_{m,n;\sigma} + c^\dagger_{m,n;\sigma} d_{m,n;\sigma} + H.c.\right)\\ &- t_{C1-C2} \sum_{m,n;\sigma} \left(a^\dagger_{m,n;\sigma}(e_{m,n;\sigma} + f_{m,n-1;\sigma}) + H.c.\right)\\ &- t_{C1-C2} \sum_{m,n;\sigma} \left(b^\dagger_{m,n;\sigma}(e_{m-1,n;\sigma} + f_{m,n;\sigma}) + H.c.\right)\\ &- t_{C1-C2} \sum_{m,n;\sigma} \left(c^\dagger_{m,n;\sigma}(e_{m,n;\sigma} + f_{m,n;\sigma}) + H.c.\right)\\ &- t_{C1-C2} \sum_{m,n;\sigma} \left(d^\dagger_{m,n;\sigma}(e_{m,n+1;\sigma} + f_{m+1,n;\sigma}) + H.c.\right) ,\end{aligned} \tag{2}$$

where e and f denote the lower and upper red atom in the unit cell of Fig. 1a) of the main text. The four sp^2-hybridized atoms are defined in Fig. 1b) of the main text.

The onsite Hamiltonian given by

$$\mathscr{H}_C = \sum_{\substack{m,n;\sigma\\ g=a,b,c,d,e,f}} E_g n^g_{m,n;\sigma} , \tag{3}$$

with $E_g = E_{C1}$ for $g = e, f$ and $E_g = E_{C2}$ for $g = a,b,c,d$ and $n^g_{m,n;\sigma} = g^\dagger_{m,n;\sigma} g_{m,n;\sigma}$ for $g = a,b,c,d,e,f$.

We also need to introduce a Hubbard term

$$\mathscr{H}_H = U \sum_{\substack{m,n\\ g=a,b,c,d}} n^g_{m,n;\uparrow} n^g_{m,n;\downarrow} \tag{4}$$

and an assisted hopping contribution

$$\begin{aligned}\mathscr{H}_a = & W\sum_{m,n;\sigma} n^a_{m,n;\bar\sigma}\left(a^\dagger_{m,n;\sigma}(e_{m,n;\sigma}+f_{m,n-1;\sigma})+H.c.\right)\\ & +W\sum_{m,n;\sigma} n^b_{m,n;\bar\sigma}\left(b^\dagger_{m,n;\sigma}(e_{m-1,n;\sigma}+f_{m,n;\sigma})+H.c.\right)\\ & +W\sum_{m,n;\sigma} n^c_{m,n;\bar\sigma}\left(c^\dagger_{m,n;\sigma}(e_{m,n;\sigma}+f_{m,n;\sigma})+H.c.\right)\\ & +W\sum_{m,n;\sigma} n^d_{m,n;\bar\sigma}\left(d^\dagger_{m,n;\sigma}(e_{m,n+1;\sigma}+f_{m+1,n;\sigma})+H.c.\right)\ , \end{aligned} \tag{5}$$

where $\bar\sigma$ is the opposite spin-projection of σ.

The interaction terms are most easily treated within the mean-field approximation. For the Hubbard interaction, we set

$$\mathscr{H}_H \approx U\sum_{\substack{m,n;\sigma\\ g=a,b,c,d}} n^g_{m,n;\sigma}\langle n^g_{m,n;\bar\sigma}\rangle + E_U\ , \tag{6}$$

where the constant energy shift reads $E_U = -U\sum\langle n^g_{m,n;\uparrow}\rangle\langle n^g_{m,n;\downarrow}\rangle$ with the sum over m,n and $g=a,b,c,d$. The assisted hopping term is approximated analogously by the following:

$$\begin{aligned}\mathscr{H}_a = & W\sum_{m,n;\sigma}\langle n^a_{m,n;\bar\sigma}\rangle\left(a^\dagger_{m,n;\sigma}(e_{m,n;\sigma}+f_{m,n-1;\sigma})+H.c.\right)+\langle n^b_{m,n;\bar\sigma}\rangle\left(b^\dagger_{m,n;\sigma}(e_{m-1,n;\sigma}+f_{m,n;\sigma})+H.c.\right)\\ & +W\sum_{m,n;\sigma}\langle n^c_{m,n;\bar\sigma}\rangle\left(c^\dagger_{m,n;\sigma}(e_{m,n;\sigma}+f_{m,n;\sigma})+H.c.\right)+\langle n^d_{m,n;\bar\sigma}\rangle\left(d^\dagger_{m,n;\sigma}(e_{m,n+1;\sigma}+f_{m+1,n;\sigma})+H.c.\right)\\ & +W\sum_{m,n;\sigma} n^a_{m,n;\bar\sigma}\left\langle a^\dagger_{m,n;\sigma}(e_{m,n;\sigma}+f_{m,n-1;\sigma})+H.c.\right\rangle + n^b_{m,n;\bar\sigma}\left\langle b^\dagger_{m,n;\sigma}(e_{m-1,n;\sigma}+f_{m,n;\sigma})+H.c.\right\rangle\\ & +W\sum_{m,n;\sigma} n^c_{m,n;\bar\sigma}\left\langle c^\dagger_{m,n;\sigma}(e_{m,n;\sigma}+f_{m,n;\sigma})+H.c.\right\rangle + n^d_{m,n;\bar\sigma}\left\langle d^\dagger_{m,n;\sigma}(e_{m,n+1;\sigma}+f_{m+1,n;\sigma})+H.c.\right\rangle + E_W, \end{aligned} \tag{7}$$

where the constant energy shift reads $E_W = -W\sum\langle n^g_{m,n;\bar\sigma}\rangle\langle\xi\rangle$ with the sum over $m,n;\sigma;g=a,b,c,d$ and $\xi = C1^\dagger C2 + H.c.$ denoting all the different hopping processes between the C1- and C2-atoms which are proportional to the hopping amplitude t_{C1-C2}.

For a half-filled band, we have $\langle n^g_{m,n;\sigma}\rangle = 1/2$ leading to the following approximation:

$$\mathscr{H}_C+\mathscr{H}_H+\mathscr{H}_a \approx E_X\sum_{m,n;\sigma}\left(n^e_{m,n;\sigma}+n^f_{m,n;\sigma}\right)+\widetilde{\mathscr{H}_0}+\sum_{m,n;\sigma}\widetilde{E}_W\ , \tag{8}$$

where we have $E_X = E_{C1}-E_{C2}-U/2-W\langle\xi\rangle/8$ and $\widetilde{E}_W = -W\langle\xi\rangle/4$. We have also set $E_{C2}=0$. The assisted hopping Hamiltonian further leads to a renormalized hopping amplitude $\tilde t_{C1-C2} = t_{C1-C2}+W/2$ and $\widetilde{\mathscr{H}_0}$ is thus obtained by replacing t_{C1-C2} by $\tilde t_{C1-C2}$ in Eq. (2).

The expectation value of the hopping processes is obtained self-consistently as $\langle\xi\rangle = 2.94$. For typical parameters, we have $U\approx 10$eV, $W\approx 2$eV, $E_{C1}\approx 2$eV and set $E_{C2}=0$ which yields $E_X\approx -3.75$eV and $\widetilde{E}_W\approx -1.5$eV. We further set $t_{C1-C2} = 0.35t_0 \approx 1$eV which yields $\tilde t_{C1-C2}\approx 2$eV.

Within this mean-field approximation, the two spin-projections decouple and we will drop the spin-degree of freedom. We are now in the position to reduce the resulting 6-band model to an effective 4-band model by projecting out the C1-atoms. This results in the effective Hamiltonian of Eq. (1) of the main text with $t\to\frac{\tilde t^2_{C1-C2}}{E_X-E}$ and $E_0 = 2\frac{\tilde t^2_{C1-C2}}{E_X-E}$. With an additional shift of $E^c_0 = 1.15$eV, the two conduction bands can be well approximated with the above parameters.

The electronic density of the two valence bands is more located between the C2-atoms than the one of the two conductance band. Also self-energy corrections are usually more dominant for the bands further away of the Fermi level, i.e., in our case the conduction band. The two valence bands are thus calculated by setting $U=W=0$. The larger energy shift of $E^v_0 = 2.61$eV needed to fit the data matches well with the predicted value $E^c - E^v \approx \widetilde{E}_W$. The four bands are shown as blue curves in Fig. 3 of the main text.

We note that the hopping Hamiltonian $\mathscr{H}_0$ has already been discussed in the Supplementary Information S3 of Ref.[3] Here, we showed that it is crucial to also include the onsite, Hubbard and assisted hopping terms in order to reproduce the band structure of the bands close to half-filling.

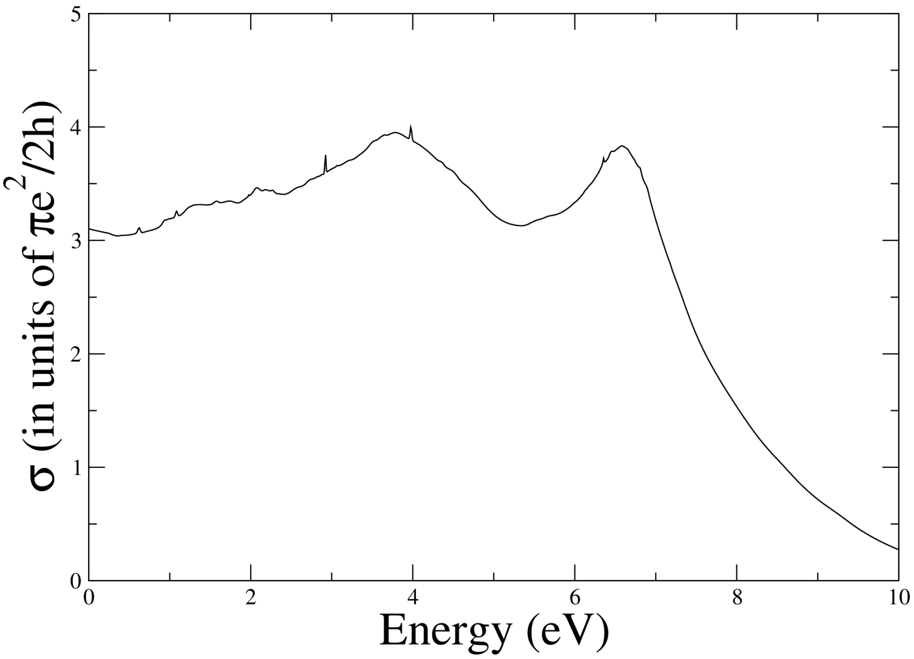

Figure 1. Optical conductivity as a function of the energy as obtained from first principles using the *Wannier90* software[4] on top of the VASP DFT calculations.

Optical absorption obtained from first principles

In order to compare the results for the optical absorption obtained using the effective Hamiltonian, we will here perform the calculations using the *Wannier90* software[4] on top of the VASP DFT calculations. By this, we can also verify the quality of the fit of the band structure based on our effective Hamiltonian.

The *Wannier90*-code allows for an effective approach to construct maximally localized Wannier functions (MLWF) from the plane wave expansion employed with the VASP code.[5] Furthermore, within the MLWF description, the *Wannier90*-code can provide the three-dimensional (3D) optical conductivity using the Kubo-Greenwood equation. In order to obtain the 2D conductivity for a penta-graphene unit cell (6 C atoms) in a 3D vector set, we have to multiply the conductivity by the norm of the perpendicular vector of the unit cell which has been chosen to be 20Å.

The final result is express in units of the universal conductivity $\sigma_0 = \frac{\pi}{2}\frac{e^2}{h}$, the conductivity of neutral single-layer graphene. The corresponding absorption is then obtained by $\mathscr{A}_0 = \frac{\sigma_0}{\varepsilon_0 c} = \pi\alpha$, with α the fine-stucture constant. Let us finally note that the results are isotropic and that we will thus not distinguish between the different directions.

The optical conductivity $\sigma(\omega)$ depends on the uniform interpolation k-point mesh parameter of *Wannier90*. After convergence to a 48x48x1 k-mesh, σ shows a plateau region of value $3\sigma_0$, with two maxima reaching $4\sigma_0$, as shown in Fig. 1. The obtained maximum values within the *Wannier90* toolkit agree well with those observed in the main text using the tight-binding approach, which related the high absorbance of penta-graphene to its chemical structure.

References

1. Hirsch, J. E. & Marsiglio, F. Hole superconductivity in oxides: A two-band model. *Phys. Rev. B* **43**, 424–434 (1991).
2. Hirsch, J. E. Electron- and hole-hopping amplitudes in a diatomic molecule. *Phys. Rev. B* **48**, 3327–3339 (1993).
3. Zhang, S. *et al.* Penta-graphene: A new carbon allotrope. *Proc. Natl. Acad. Sci. U. S. A.* **112**, 2372–2377 (2015).
4. Mostofi, A. A. *et al.* A Tool for Obtaining Maximally-Localised Wannier Functions. *Comput. Phys. Commun.* **178**, 685 (2008).
5. Souza, I., Marzari, N. & Vanderbilt, D. Maximally localized Wannier functions for entangled energy bands. *Phys. Rev. B* **65**, 035109 (2001).
